# Effect of cryopreservation on delineation of immune cell subpopulations in tumor specimens as determinated by multiparametric single cell mass cytometry analysis

**DOI:** 10.1186/s12865-017-0192-1

**Published:** 2017-02-02

**Authors:** Elma Kadić, Raymond J. Moniz, Ying Huo, An Chi, Ilona Kariv

**Affiliations:** 1 Department of Pharmacology, Cellular Pharmacology, Merck and Co. Inc, 33 Avenue Louis Pasteur, Boston, 02115 MA USA; 2 Department of Biology-Discovery, Immunooncology, Merck and Co. Inc, Boston, MA USA; 3 Department of Chemistry, Capabilities Enhancement, Merck and Co. Inc, Boston, MA USA

**Keywords:** Mass Cytometry (MC), CyTOF™, Flow cytometry (FC), Tumor samples, Tumor microenvironment (TME), Tumor infiltrating lymphocytes (TILs), Immunophenotyping, Cryopreservation, ViSNE

## Abstract

**Background:**

Comprehensive understanding of cellular immune subsets involved in regulation of tumor progression is central to the development of cancer immunotherapies. Single cell immunophenotyping has historically been accomplished by flow cytometry (FC) analysis, enabling the analysis of up to 18 markers. Recent advancements in mass cytometry (MC) have facilitated detection of over 50 markers, utilizing high resolving power of mass spectrometry (MS). This study examined an analytical and operational feasibility of MC for an in-depth immunophenotyping analysis of the tumor microenvironment, using the commercial CyTOF™ instrument, and further interrogated challenges in managing the integrity of tumor specimens.

**Results:**

Initial longitudinal studies with frozen peripheral blood mononuclear cells (PBMCs) showed minimal MC inter-assay variability over nine independent runs. In addition, detection of common leukocyte lineage markers using MC and FC detection confirmed that these methodologies are comparable in cell subset identification. An advanced multiparametric MC analysis of 39 total markers enabled a comprehensive evaluation of cell surface marker expression in fresh and cryopreserved tumor samples. This comparative analysis revealed significant reduction of expression levels of multiple markers upon cryopreservation. Most notably myeloid derived suppressor cells (MDSC), defined by co-expression of CD66b^+^ and CD15^+^, HLA-DR^dim^ and CD14^−^ phenotype, were undetectable in frozen samples.

**Conclusion:**

These results suggest that optimization and evaluation of cryopreservation protocols is necessary for accurate biomarker discovery in frozen tumor specimens.

**Electronic supplementary material:**

The online version of this article (doi:10.1186/s12865-017-0192-1) contains supplementary material, which is available to authorized users.

## Background

Cancer represents a multifaceted disease characterized not only by extreme genetic and epigenetic heterogeneity of the transformed cells, but also by clonal progression of treatment-resistant tumors as a result of targeted therapies. In addition to molecular resistance, tumor-mediated suppression of the self-immune system is thought to contribute to tumor evasion from effector cells [1]. Early efforts to modulate immune system’s anti-tumor functionality has resulted in only marginal therapeutic efficacy [2, 3]. However, to date, more than 10 antibody (Ab)-based therapies are available in the clinic [4]. Most recent are targeting two immuno-checkpoint receptors; CTLA4 (cytotoxic T-lymphocyte-antigen 4), PD-1 (programmed cell death 1) and its ligand PDL1, and show a remarkable efficacy against certain tumors, through re-activating cytotoxic T (Tc) cell mediated tumor killing [5, 6]. These positive clinical outcomes support further investment in targeting additional immunomodulatory receptors on T-cytotoxic (Tc) cells, and expanding these approaches to other immune cells. For example natural killer (NK) cells can be recruited to kill tumor cells, without potential concern for causing acute cytokine storm or long-term autoimmune responses [7, 8], while suppression of T-regulatory (Treg) cells can be used to enhance immune response against tumor cells [9].

In order to accelerate the delivery of immunomodulators from the bench to the bedside, it is imperative to have a toolbox of biomarkers that can be used for in-depth understanding of the complexity of disease-underlying biology, and refine translational validity between preclinical efficacy and the highly diverse patient response [10–12]. To address this, a greater emphasis on the use of clinical specimens for discovery of disease relevant biomarkers has been placed for diagnosis, prognosis, assessment of treatment efficacy and patient stratification strategies [13, 14]. Today, the collection of patient samples, such as core-needle biopsies and blood, for genetic and other analyses has become common practice, offering accurate determinations from limited clinical tissues [15]. However, effective bioanalytical tools that encompass complex immune- and tumor cells interactions in a clinical trial setting have been lacking [16, 17].

For the past several decades multiparametric fluorescent cytometry has been used in research and clinical laboratories to significantly advance biomarker discovery by immunophenotyping highly heterogeneous tumor samples [18–20]. However due to spectral overlap of fluorophore conjugated to detection antibodies, and inherent high sample auto-fluorescence, practical detection is currently limited to 12–18 simultaneous independent markers [21–23]. The recently developed mass-spectrometry (MS) based technology, or commonly referred to as cytometry by time-of-flight (CyTOF™), has enabled multiplexed cellular analysis of up to 100 parameters. Mass cytometry has overcome many limitations seen with FC, by utilizing metal conjugated antibodies coupled with atomic MS detection [24]. The use of rare-earth transitional metals as detection tags provides a clear advantage over fluorescent labels as these are not naturally occurring within the human body, and the added advantage of MS resolution capabilities of single mass differences, allows for quantification of signals without cellular background interference or significant signal spillover, thus making CyTOF™ well suited to for multi-dimensional single cell analysis of limited clinical specimens [25, 26]. Furthermore, integration of highly multiplexed detection on a single cell level with an advanced statistical analysis allows for the un-biased delineation of the cellular subsets that can be easily overlooked by trying to assemble several detection Ab panels utilized by flow cytometry [27]. In the short period of time since the introduction of this technology several break-through studies demonstrated the potential of this platform to interrogate complex mechanistic and biomarker networks [28–30]. Moreover, recent studies of tumor samples demonstrated that MC could greatly improve knowledge of the complex cellular milieu of acute myeloid leukemia (AML) by utilizing 35–40 simultaneous detection markers [31]. While findings like these have highlighted the advanced analytic capabilities of MC, the use of this platform to develop a fit-for-purpose immunophenotyping analysis of tumor specimens from clinical trials remain to be evaluated in the context of clinical sample handling logistics.

In this study, MC detection stability was first evaluated by nine independent experimental runs of a well-characterized single PBMC lot utilizing 14 cell surface markers. After establishing optimized MC throughput, detection and data analysis protocols, we expanded on previous publications [32, 33] by further validating applicability of the comprehensive immunophenotyping of PBMCs and clinical tumor samples as compared to measurements obtained using conventional FC. The cumulative findings of these studies confirmed that the CyTOF™ platform supports comprehensive multiparametric biomarker discovery, as evidenced by the analysis of 39 simultaneously detected markers.

While assessing accuracy of the high multiparametric analytical potential of MC detection, this study further evaluated the impact of cryopreservation on the immune cell markers by comparing fresh and subsequently cryopreserved human tumor specimens. Due to concerns about clinical sample stability during shipment and in an effort to standardize analysis conditions across many specimens, the samples are commonly cryopreserved shortly after collection [34], and analyzed at a later time as a single batch to minimize technical and systems variability. However recent publications have raised questions about sample integrity and reliability of these specimens [35, 36]. Results of our study showed a significant reduction in expression levels of most myeloid markers such as CD11B, CD14, CD15, CD16, CD66, CD86, CD80, and CD56 as well as immunoregulatory receptors (IMRs) upon cryopreservation. Most notable was the complete loss of detection of myeloid derived suppressor cells (MDSC). These results strongly caution the use of cryopreserved tumor samples for biomarker discovery and merit further studies to identify advanced cryopreservation protocols.

## Methods

### Fluorescent and mass cytometry detection antibodies

#### Sample sourcing

PBMCs were purchased from SeraCare Life Sciences Inc., (Milford, MA). Frozen dissociated tumor cells (DTC) and normal adjacent tissue (NAT) were purchased from ConversantBio (Huntsville, AL). The frozen samples were shipped on dry ice and stored in liquid nitrogen. Dissociation and cryopreservation of tumor tissue specimens, resected in 2010 and 2012, were performed by the vendor by applying tissue specific protocols that employed both enzymatic and mechanical dissociation. Fresh human tumor specimens were properly collected with all necessary approvals, consents and/or authorizations for the collection, use and/or transfer of such human tissues through Neurologica Cognitiva Research LLC DBA Boston Biosource (Newton, MA). Tumor samples were stored in AQIX® media (AQIX LTD, London, UK), a formulation optimized to maintain pH levels and mimic the intestinal fluid layer while preserving genetic and histological profiles of excised tissue for up to 72 h following removal from patients [37]. The samples were shipped and stored at 4 °C, and tissue processing occurred within 12 h of surgical removal.

#### Frozen sample recovery

Cells were rapidly thawed in a 37 °C water bath, and diluted in pre-warmed complete medium: Roswell Park Memorial Institute (RPMI) 1640 medium supplemented with 10% fetal bovine serum (FBS), (both LifeSciences, Carlsbad, CA). Residual dimethyl sulfoxide (DMSO) was removed by centrifugation at 400 g for 5 min and pelleted cells were resuspended in growth media and allowed to recover for 30 min at 37 °C and 5% CO_2_ prior to subsequent procedures [38].

#### Fresh tissue dissociation

Dissociation of fresh renal cell carcinoma and colorectal tumor tissues was performed according to manufacturer’s instructions for the human tumor dissociation kit (Milteny, Auburn, CA); briefly the tissue samples were cut up into smaller pieces and subjected to enzymatic and mechanical dissociation using the vendor supplied enzyme cocktail and the GentleMACS™ dissociator (Miltenyi, Auburn, CA). The mechanical dissociation protocol employed variable blade rotation speeds for 60 min at 37 °C. After tissue dissociation, red blood cells (RBC) were lysed using Ammonium-Chloride-Potassium (ACK) buffer (Life Sciences, Carlsbad, CA) for 5 min at RT. The samples were washed twice with complete medium by pelleting at 400 g for 5 min and the cell count was determined using Vi-Cell (Beckman Coulter, Indianapolis, IN). The resulting single sell suspension was stained for immediate analysis by FC and MC while residual cells were cryopreserved.

#### Cryopreservation of dissociated tumor cells (DTC)

The cells were cryopreserved in four different freezing media at concentrations ranging from 2 × 10^6^ to 5 × 10^6^ cells/mL. The following cryopreservation media (CM) were tested; CM1: 90% FBS (Gibco, Grand Island, NY) and 10% DMSO Hybri-Max™ (Sigma-Aldrich, St. Louis, MO); CM2: 50% AQIX^®^ media, 40% FBS and 10% DMSO Hybri-Max™; CM3: 90% AQIX media and 10% UltraPure™ Glycerol (Invitrogen, Carlsbad, CA); CM4: CryoScarless DMSO-Free media (BioVerde, Kyoto, Japan). The freezing media was added gently to the cells and transferred to sterile Nalgene® cryogenic vials (Sigma-Aldrich, St. Louis, MO). CoolCell® alcohol-free cell freezing containers (Biocision, San Rafael, CA) were used to limit rate of freezing to a −1 °C to −3 °C per minute temperature drop. After 24 h incubation at −70 °C, the cryovials were transferred to −140 °C liquid nitrogen for long term storage. Two cryovials from each cryomedia formulation were recovered after 28 and 56 days for immunophenotyping analysis.

#### Flow cytometry staining and acquisition

Cells were stained for FC via traditional methods. Briefly, cells were re-suspended in Dulbecco's Phosphate Buffered Saline (DPBS) (GE Healthcare, Logan, UT) and stained with cell viability dye (Life Technologies, Carlsbad, CA) for 15 min on ice. Cells were washed twice by pelleting at 400 g for 5 min, using standard FC buffer (1% BSA (w/v) in DPBS). The samples were then treated with human Fc Block™ (BD Biosciences, Franklin Lakes, NJ) for 15 min at a concentration of 2.5 μg per 1 × 10^6^ cells in FC buffer. The samples were washed once, and incubated for 60 min on ice with the antibody cocktail prepared in FC buffer. Following incubation the samples were washed twice with FC buffer and analyzed using LSR Fortessa SORP (BD Biosciences, Franklin Lakes, NJ). Compensation was performed using AbC™ bead kit (Invitrogen, Carlsbad, CA) and fluorescence minus multiple (FMM) controls were employed to benchmark sample background and signal-spillover. A high-throughput sampler (HTS) module was used for sample acquisition.

#### Mass cytometry staining and acquisition

The cells were stained as previously described [28]. Briefly, in preparation for staining with Lanthanide-conjugated antibodies, the samples were resuspended and incubated for 30 min at 37 °C with Cell-Staining-Medium (CSM) and 1X ^103^Rh DNA Intercalator (both Fluidigm, San Francisco, CA) at a concentration of 1 × 10^6^ viable cells/mL. The samples were pelleted by centrifugation at 400 g for 5 min at RT and incubated for 20 min at RT with 10 μl of human TruStain FcX™ (BioLegend, San Diego, CA). A mixture of antibodies, using vendor specified concentrations as well as concentrations determined by single stain titration (data not shown), in CSM, was added to a final volume of 100 μl/well. The samples were incubated with staining antibodies for 60 min at 4 °C with gentle vortexing. The samples were washed twice and incubated for 60 min at 4 °C with Fix/Perm buffer containing 1x ^191,193^Ir DNA Intercalator (both Fluidigm, San Francisco, CA), and then again twice with DPBS and resuspended in Milli-Q® water (Millipore, Billerica, MA). The samples were acquired using CyTOF™, with upgraded mass channel range (CyTOF™ 2, Fluidigm, San Francisco, CA), as previously described [24, 29]. Metal minus multiple (MMM) control samples were used to define positive signals and determine spillover, if any. Daily maintenance and tuning was performed according to manufacturer’s instructions [39]. In addition to internal vendor-set calibration procedures, Europium beads were incorporated into daily operation before and after sample analysis, enabling inter-run normalization.

#### Data analysis

Flow cytometry samples were analyzed using FCS Express 4 Flow RUO (De Novo Software, Glendale, CA). CyTOF™ data was analyzed using Cytobank (Cytobank, Inc., Mountain View, CA) as previously described [40]. Briefly doublets and debris were excluded from analysis using previously described gating schemes [41], and manual gating on bivariate plots allowed for identification of populations of interest using published phenotypes [42]. Similarly exclusion of doublets and debris from FC data was performed using traditional methods in published literature. Positive signals were identified using FMM and MMM controls as well as anti-CD3/CD28 activated PBMCs overexpressing immunoregulatory receptors (IMRs) (data not shown). Pearson product moment correlation (PPMC) was used to compare FC and MC data sets, with a two tailed p value (GraphPad Software V.6, La Jolla, CA). The data sets were compared using median intensity values measured for common antibodies. Non-hierarchical, clustering of tumor samples was performed using viSNE [43]. Briefly, high-dimensional biological data generated by mass cytometry is reduced to two dimensions using the Barnes-Hut implementation of the t-SNE algorithm [44], and visualized as a traditional scatter plot. Between 10,000 and 40,000 cells were sampled and live singlet cells were used as the parent population, subsequent clustering was performed using markers included in the panel. Expression levels were displayed as median intensities in all viSNE plots.

## Results

### Determination of mass cytometry inter-run reproducibility

**Fig. 1:**
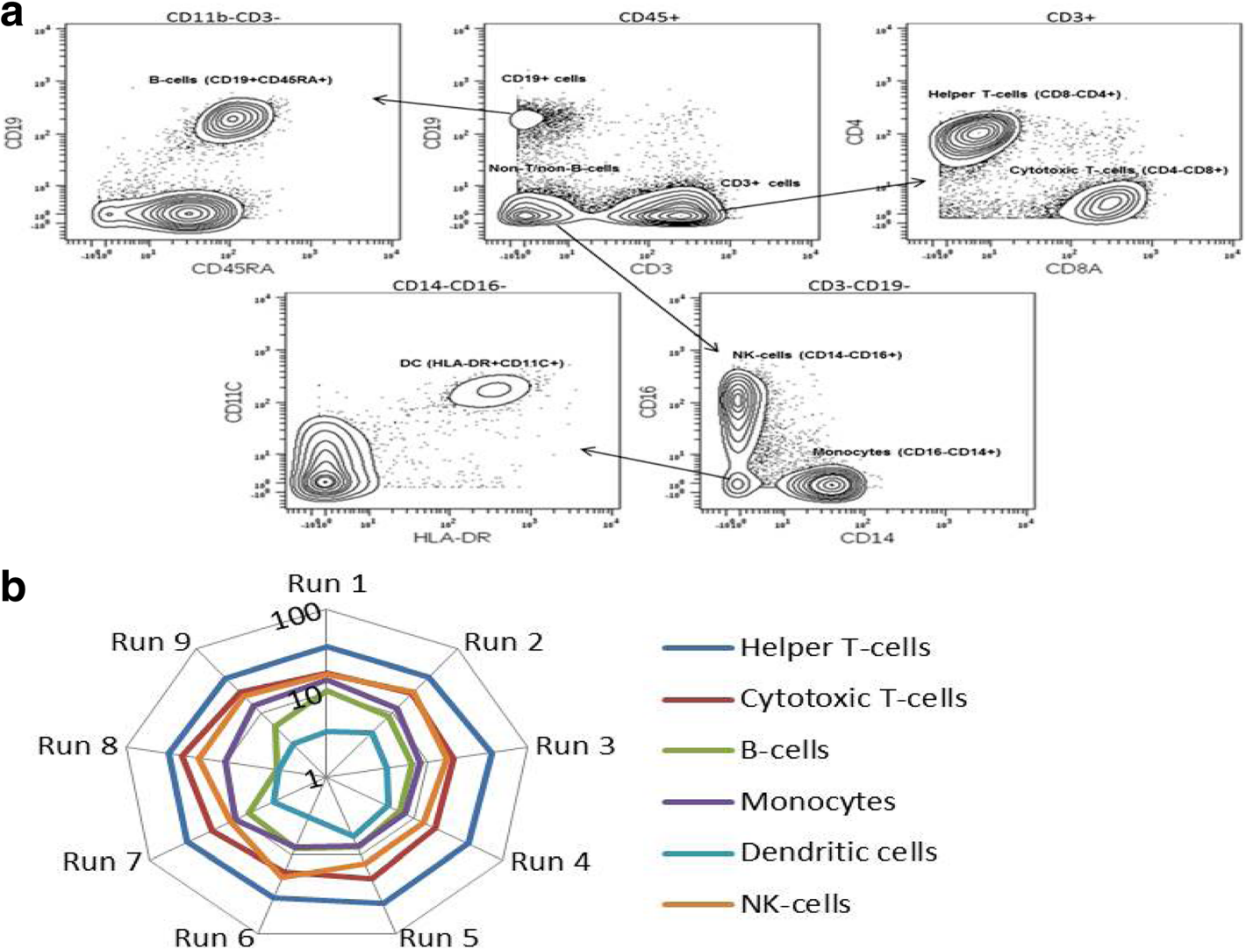
Determination of inter-run reproducibility of mass cytometry platform (CyTOF2™). **a** Representative gating scheme applied to identification of immune subsets in PBMCs from a single donor. **b** Longitudinal analysis of major immune subsets in CD45+ cells (*N* = 9). Data is expressed as percentage of CD45+ cells on log_10_ scale

A commercially sourced lot of frozen PBMCs from a single healthy donor were used to optimize CyTOF™ protocols, and to establish inter-assay variability for mass cytometry as assessed by measurements of well-defined immune cell subsets. For all experiments a total of two-million cells were stained per sample. Due to cell loss during sample preparation and CyTOF™ sampling [45], on average 300,000 to 500,000 cells were acquired per run. By adhering to published guidelines for rare-event analysis [46] we chose to exclude subpopulations consisting of less than 100 cellular events. The gating scheme used to identify major cellular subsets is shown in Fig. 1a. Dead cells and doublets were excluded from analysis using previously described methods for MC and FC [41, 47, 48]. CD45 positive cells were used as the parent population for the initial bivariate plot identifying T-cells (CD3+) and B-cells (CD19+). The T-cell compartment was further delineated into helper T-cells (CD3 + CD4+) and cytotoxic T-cells (CD3 + CD8+), whereas B-cell were identified by co-expression of CD19+ and CD45RA+. The CD3-CD19- double negative population was used to identify monocytes (CD14+) and NK-cells (CD14-CD16+), while the dendritic cell (DC) phenotype was characterized by the absence of CD16- and CD14- expression, and positive co-expression of CD11c and HLA-DR. The Log_10_ radial plot (Fig. 1b) summarizes the distribution of these cellular subsets across nine independent assays. Average values for helper-T-cells, cytotoxic-T-cells and NK-cells represented 38.1 ± 3.2, 20.0 ± 3.4 and 16.3% ± 3.1% of CD45^+^ cells respectively, while B-cells, monocytes and MDCs made up a smaller fraction of PBMCs with 7.4 ± 2.1, 10.3 ± 2.5 and 4.1% ± 1.0% respectively. This data confirms significant inter-run correlation using MC detection, across nine independent runs.

### Comparison of MC and FC for frozen PBMC and DTC sample analyses

**Fig. 2:**
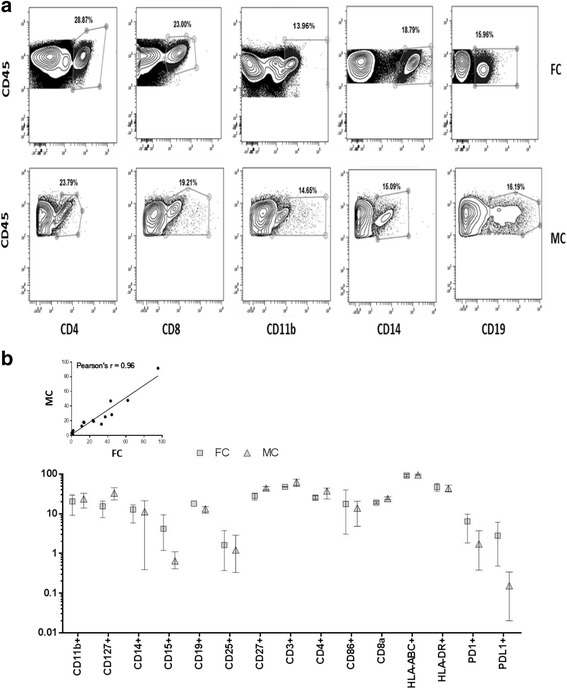
Detection of cellular subsets in PBMC samples by mass and fluorescent cytometry. **a** Representative gating scheme identifying major immune cell populations in PBMCs by FC and MC. Singlet cells, deemed viable by a Live/Dead marker (FC) or DNA intercalator (MC) were used as the parent population for cell surface marker analysis. Percentage of positive cells on a bivariate plot of CD45 and markers common to both platforms were compared. *Markers* included in analysis: CD11b, CD127, CD14, CD15, CD19, CD25, CD27, CD3, CD4, CD86, CD8a, HLA-ABC, HLA-DR, PD-1 and PD-L1. **b** Comparison of population percentages quantified by FC and MC. Percentages of cells positive for CD45 and *15 common markers* were quantified by both platforms. Data represents log_10_ (average) ± standard deviation (SD) (*N* = 3) of percent positive cells. Correlation between FC and MC was determined by Pearson Product Moment Correlation (PPMC) (*r* = 0.96, *p* < 0.0001)

To further confirm accuracy of mass cytometry detection as compared to flow cytometry, we used the same lot of frozen PBMCs to compare frequencies of cellular subtypes, as determined by both detection methods. The PBMC samples were stained for common cell surface markers, with clonally matched Abs whenever possible for both platforms (Table 1). The number of detection markers was limited to one panel of 15 Abs due to the FC detection limitations [21]. The percentage of positive cells on the bivariate plot of CD45+ and markers common to both platforms was measured in three independent experiments (Fig. 2a). The results obtained using FC and MC detection were compared using PPMC analysis (Fig. 2b), and data indicated a statistically significant agreement in percentage distributions across both platforms as evident by the correlation coefficient (r) value of 0.96 (*p* < 0.0001). These findings are in agreement with previously published reports indicating comparable results between fluorescent and mass cytometry for PBMCs [31, 33].

**Table 1:** Antibody Panels for Flow and Mass Cytometry Analyses

Antibody	Clone		Conjugate for detection		Vendor	
	FC	MC	FC	MC	FC	MC
CD3	UCHT1	UCHT1	BUV737	170Er	BD	FL
CD4	RPA-T4	RPA-T4	PerCPCy5.5	145Nd	BL	FL
CD8a	RPA-T8	RPA-T8	AF 700	146Nd	BL	FL
CD11b	ICRF44	ICRF44	APC-Cy7	144Nd	BL	FL
CD11c	Bu15	Bu15	AF 488	147Nd	BL	FL
CD14	HCD14	M5E2	BV 737	160Gd	BD	FL
CD15	W6D3	W6D3	BV 605	164Dy	BD	FL
CD16	n/a	3G8	n/a	148Nd	n/a	FL
CD19	HIB19	HIB19	AF 800	142Nd	BL	FL
CD25	2A3	2A3	PE	169Tm	BD	FL
CD27	L128	L128	BV 510	155Gd	BD	FL
CD33	n/a	WM53	n/a	158Gd	n/a	FL
CD38	n/a	HIT2	n/a	167Er	n/a	FL
CD44	n/a	BJ18	n/a	166Er	n/a	FL
CD45	HI30	HI30	BUV395	154Sm	BD	FL
CD45RA	n/a	HI100	n/a	143Nd	n/a	FL
CD45RO	n/a	UCHL1	n/a	149Nd	n/a	FL
CD56	HCD56	HCD56	PE-Cy7	176Yb	BL	FL
CD62L	n/a	DREG-56	n/a	153Eu	n/a	FL
CD66b	G10F5	80H3	AF 647	152Sm	BD	FL
CD66	n/a	CD66a-B1.1	n/a	171Yb	n/a	FL
CD80	L307.4	n/a	BV 510	n/a	BD	n/a
CD86	2331	IT2.2	BV 510	156Gd	BD	FL
CD107a	n/a	H4A3	n/a	151Eu	n/a	FL
CD127	A019D5	A019D5	BV 605	165Ho	BL	FL
CD152	n/a	14D3	n/a	161Dy	n/a	FL
CD183	n/a	G025H7	n/a	156Gd	n/a	FL
CD185	n/a	51,505	n/a	171Yb	n/a	FL
CD194	n/a	205,410	n/a	158Gd	n/a	FL
CD196	n/a	G034E3	n/a	141Pr	n/a	FL
CD197	n/a	G043H7	n/a	159 Tb	n/a	FL
CD223	n/a	874,501	n/a	150Nd	n/a	FL
CD273	MIH18	24 F.10C12	BV 711	172Yb	BD	FL
CD274	29E.2A3	29E.2A3	BV 421	175Lu	BL	FL
CD279	EH12.2H7	EH12.2H7	BV 786	175Lu	BL	FL
CD357	621	In-house	PE	159 Tb	BL	FL
TIGIT	MBSA45	n/a	AF 647	n/a	eBio	n/a
KI-67	n/a	Ki-67	n/a	168Er	n/a	FL
HLA-DR	L243	L243	PerCPCy5.5	174Yb	BL	FL
HLA-ABC	G45-2.6	W6-32	PE-Cy7	141Pr	BD	FL
FOXP3	259D	PCH101	AF 488	162Dy	BL	FL

The antibodies used in both fluorescent and mass cytometry were commercially sourced

*Abbreviations*: *MC* mass cytometry, *FC* flow cytometry, *AF* Alexa Fluor, *BV* Brilliant Violet, *BUV* Brilliant Ultra Violet, *FL* Fluidigm, *BL* BioLegend, *BD* BD Biosciences, *eBio* eBiosciences

**Fig. 3:**
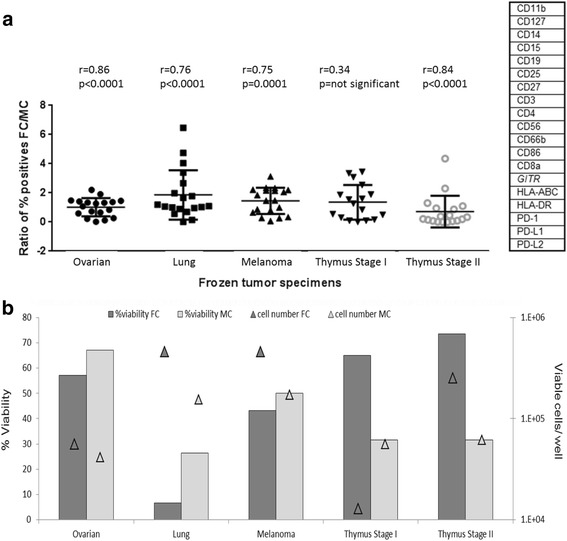
Comparison of FC and MC detection of immunophenotyping markers in frozen primary tumor samples. **a** Ratio of percent positive cells detected by FC over MC for 19 common markers. Correlation between FC and MC was determined by Pearson Product Moment Correlation (PPMC). **b** Cell number and viability measurements. Viability and cell number as determined by FC analysis using an amine-reactive dye. Viability and number of acquired events as determined by MC analysis using ^103^Rh and ^191/193^Ir DNA intercalators. Cellular counts are expressed on a log_10_ scale. Data indicate that the viability and cell number differences acquired using FC and MC were major contributing factors in divergence of detected cellular frequencies in tumor samples

In order to determine if the correlation between MC and FC observed in a single donor PBMC lot extends to significantly more heterogeneous tumor samples, comparison experiments were conducted using five frozen commercial DTCs. Upon thawing cell suspensions were split for FC and MC analysis, and subsequently further split into staining and control panels. The frequency of positive cells was measured on bivariate plots of CD45^+^ and markers common to both platforms, as detailed in the gating strategy for PBMC analysis (Fig. 2a). For the FC analysis, samples were subdivided into four panels; myeloid, lymphoid and corresponding FMM panels, while MC samples were split into a control MMM panel, and a single staining panel. Due to limited sample availability, only one comparison experiment was feasible for each tumor sample, with acquired cell numbers ranging from 13,000 to 500,000 on either platform (Fig. 3b). The distribution of ratios of percentages of positive cells determined by FC over MC, for each of the 19 common markers, is summarized in Fig. 3a. The correlation between FC and MC detection varied between specimens, with r values ranging from 0.34 to 0.86. The greatest agreement was observed for the ovarian tumor sample with frequency distribution within two-fold of each other, and the corresponding r of 0.86 and *p* <0.0001 values confirming significant correlation across both detection platforms. The greatest discrepancy in measurements was apparent for the stage I thymus sample, with the FC/MC ratios of the measured populations being greater than three-fold, *r* = 0.34. A detectable difference in percentage of measured populations was also observed between MC and FC analysis for the lung sample (*r* = 0.77), with percentages for CD15, CD86 and GITR showing between 4 and 6-fold difference in calculated ratios between platforms.

Further analysis indicated that samples with high correlation across the two platforms also had a greater percentage of viable cells, and this correlation was also significantly better in samples where starting cell numbers were similarly detected by both FC and MC (Fig. 3b). While viability determination by FC employed amine reactive dyes, which are added prior to fixation and permeabilization [49], MC utilized two distinct DNA intercalators; one which is added pror to permeabilazation (^103^Rh), and a second DNA intercalator (^191/193^Ir) which is added in a buffer containing paraformaldehyde and saponin [50]. Although fixation and permeabilization after cell surface staining is a standard procedure for immuno-staining and has minimal consequences to epitope binding, it is possible that tumor cells, already subjected to enzymatic and mechanical dissociation as well as cryopreservation are more sensitive to membrane effects imposed by even brief exposures to detergents [51]. Previous publications comparing FC-based detection of sample viability and cellular enumeration to other methods, reported that poor specimen quality and low cellularity samples generally result in inconsistencies across different methodologies [52, 53]. Thus a combination of both limited cell numbers available for analysis and the inherent differences in cell viability determination methodologies between FC and MC in these samples can partially account for discrepancies in detected positive cell percentages.

### Validation of multiparametric MC analysis for biomarker discovery

**Fig. 4:**
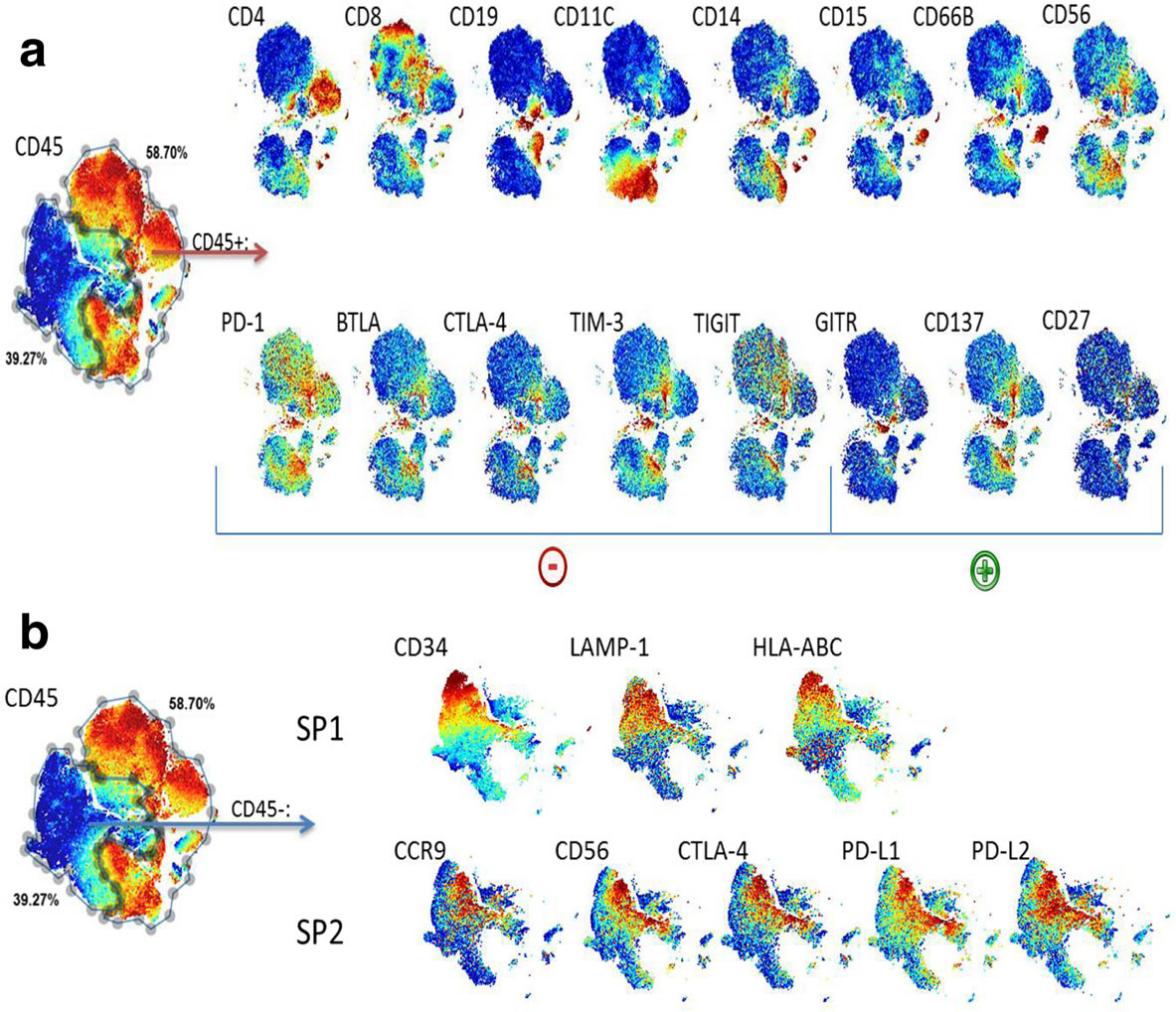
MC analysis of expression patterns of immunomodulatory and disease prognostic biomarkers in immune and tumor cell subsets. ViSNE analysis of fresh renal cell carcinoma performed using single nucleated cells as top level population. A total of 40,000 cells was analyzed and clustered using the following markers: CD45, CD19, CD11B, CD4, CD8A, CD11C, CD34, CD66B, CD14, CD15, CD3 and CD56. The cells in the ViSNE map are colored according to the median intensity of expression for markers as identified in the top left corner of each figure. **a** Immunophenotyping of CD45+ cells in solid tumor sample. T-cell subsets are identified by expression of CD4 and CD8 markers, B-cells by expression of CD19, NK cell by CD56+, DC cells by CD11C+, Monocytes CD14+, Neutrophils CD15+, and MDSC by CD15+/CD66B+. Checkpoint regulatory receptors are represented by inhibitory (PD-1, BTLA, CTLA-4, TIM-3, and TIGIT) and stimulatory (GITR, CD137, CD27) markers on identified cellular subsets. **b** Immunophenotyping of CD45- cells in solid tumor sample. Specific subsets (Sp) were identified based on these expression patterns; Sp1: CD34^bright^, CD107a^+^, HLA-ABC^+^, HLA-DR^mid^. Sp2: CCR9^+^, CD56^+^, CTLA-4^+^, PD-L1^+^, PD-L2^+^

Consistent results from MC and FC in reproducibility studies using the same sample indicate that both platforms perform similarly in quantifying common leukocyte lineage markers in both PBMC and tumor samples. However, increased multiplexing potential of the MS detection can significantly facilitate the discovery of specific immune cell subsets involved in mediating anti-tumor activity [54, 55]. Applicability of this platform to biomarker discovery and its translational value was evaluated next. A representative example of an in-depth cell population analysis of a fresh renal carcinoma (RCC) tumor enabled by MC detection was performed using a 39 surface marker CyTOF™ panel (Additional file 1). Data in Fig. 4 illustrates median expression levels of selected surface markers in tumor infiltrating lymphocytes (TILs) and identifies distinct subpopulations (Sp) in tumor cells in the RCC sample. Approximately 40,000 single nucleated cells were evaluated by a viSNE analysis [43], using CD45, CD19, CD11B, CD4, CD8A, CD11C, CD34, CD66B, CD14, CD15, CD3 and CD56 markers for the cell population clustering. We performed a typical immunophenotyping analysis (Fig. 4a) in which first distinct leukocyte (CD45+) populations are identified, and subsequently expression of both inhibitory (marked by a -) and stimulatory (marked by a +) checkpoint receptors [56] are evaluated on these subsets. Data demonstrates that T-and NK cell subsets comprise a large percentage of TILs, and indicates that inhibitory check point receptors are predominantly co-expressed by CD56 and CD8 positive cells. Our findings are in agreement with other published reports [57–59] and can be used not only in the biomarker discovery, but also benchmarking responsive patient population in the clinical settings. While immunophenotyping of solid tumors is not unique to MC, and has been reported using FC both in the research and clinical settings [19, 60], the maximum number of analytes by FC still remains well below of total of 39 markers used in our studies, as well as reported by others [61], Taking advantage of all the markers in our panel beyond identifying leukocyte phenotypes we extended this analysis to the tumor cells (Fig. 4b). Sp1 cells were marked by expression of CD34, CD107a (LAMP-1) and HLA-ABC, while Sp2 cells expressed CD199 (CCR9), PD-L2, CTLA, CD56 (NCAM), PD-L1 and PD-L2. Sp1 and Sp2 made up 39.59 and 22.44% of the tumor cells respectively. These data indicate that analysis of fresh clinical specimens using high multiplexing capabilities of CyTOF™ can provide insights into tumor cell microenvironment, thus enabling in-depth studies of complex interplay between tumor cells and infiltrating immune cells, and potentially elucidating novel targets for immunotherapy. This analysis was also applied to fresh colorectal carcinoma (CRC) specimen (Additional file 2) with similar findings.

### Effects of cryopreservation on cell viability, and lymphoid and myeloid cell lineages detection in tumor samples

The effects of cryopreservation on clinical specimens resulting in loss and/or alteration of multiple cell surface and intracellular marker detection, has long been a challenge for accurate sample immunophenotyping on different detection platforms, potentially hindering determinations of immune cell subpopulations relevant to patient stratification [62]. These changes might be due to decrease in either receptor expression levels or modifications in epitope conformation rendering these no longer accessible to detection antibodies. With the emergence of high-multiplexing detection of 50 or more markers by mass cytometry, the overall effect of cryopreservation can be now effectively interrogated on a single cell level.

**Fig. 5:**
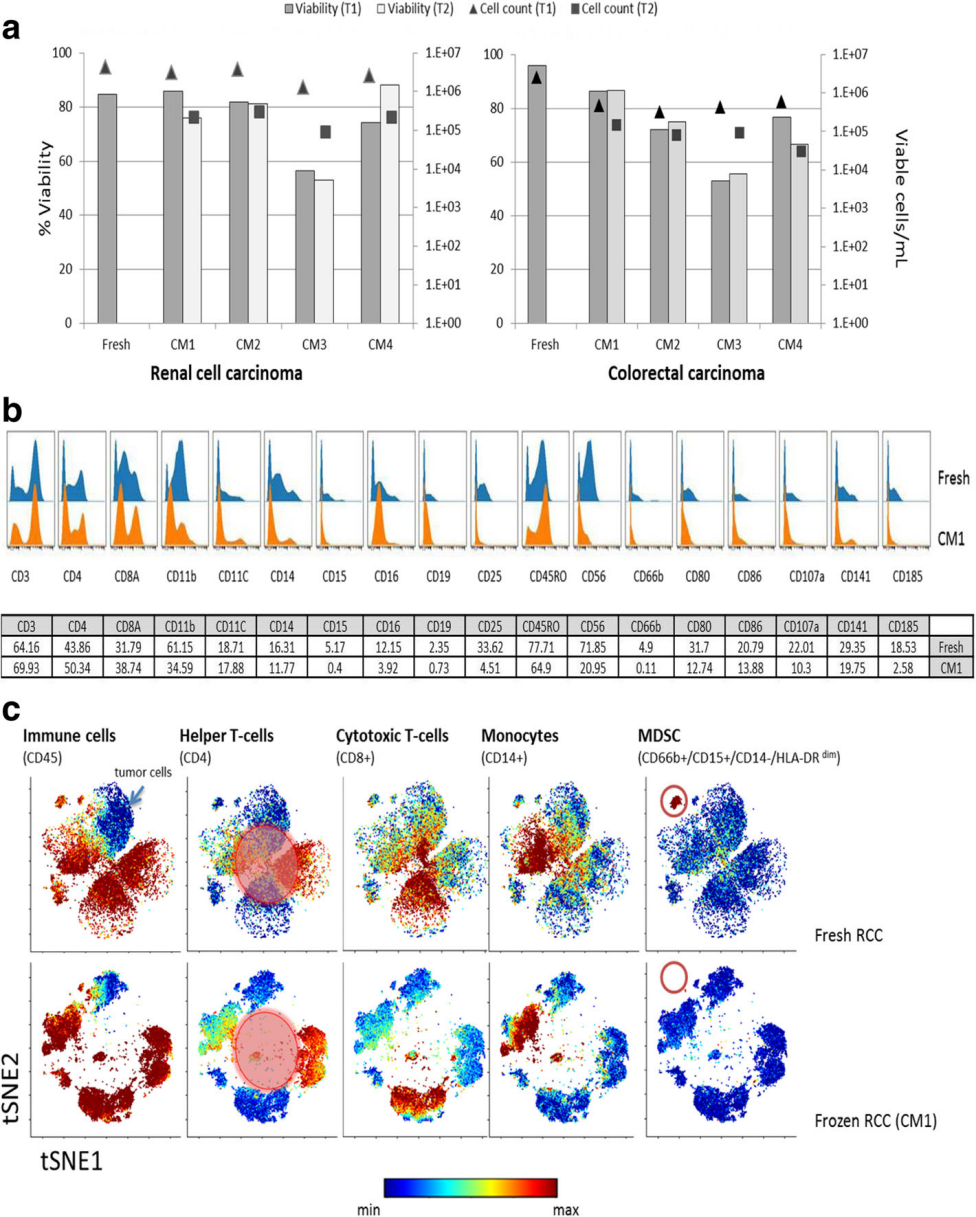
Cryopreservation effects on cellular viability and cell number recovery in renal cell carcinoma and colorectal carcinoma. **a** Viability and cell number determined by Trypan blue exclusion. For each tumor type 2–3 million cells per vial were frozen and samples were recovered in 10 mL complete media. Determinations of viable cells/mL are shown on a log_10_ scale. **b** Cryopreservation effects on surface marker detection and cellular subset identification in primary renal cell carcinoma. Comparison between fresh and CM1-cryo preserved renal cell carcinoma samples is expressed as histogram overlays of surface markers in CD45^+^ parent population. Percent positive cells as part of CD45+ population associated with selected cell surface markers are summarized in complementary table. **c** ViSNE analysis of fresh and cryopreserved renal cell specimen. The viSNE maps are colored based on median expression of selected cell surface markers, the intensity levels are represented by sliding scale. Equal sampling totaling 25,000 cells for both fresh and frozen specimens was used in this analysis. Singlet-live cells, as determined by cell length and cellular nucleation state, were used as the top level population. Clustering was done using the following markers: CD45, CD19, CD11B, CD4, CD8a, CD11C, CD34, CD66B, CD14, CD15, CD44, CD3, CD56, and CD16. CM1 formulation (T1) was selected for fresh to frozen comparison. Expression of cell surface markers in upper left corner is represented by ViSNE regions colored in gradient of red. Differences in ViSNE scatter plots between fresh and frozen specimens directly highlight differences in expression levels of surface markers used for clustering, and are highlighted by red masking

In order to determine cryopreservation effects associated with long-term storage of tumor samples, an immunophenotypic analysis of fresh tumor specimens immediately after tissue dissociation was performed and compared to the samples analyzed on day 28 (T1) and 58 (T2) after cryopreservation. For this analysis fresh primary RCC and CRC were obtained and processed within 12 h after surgical removal. The tumor specimens were greater than 2.0 g in mass. Dissociated cells were used for immediate analysis, and remaining cells were cryopreserved and used in subsequent experiments. 30 to 50 million cells were obtained per tumor sample, allowing for at least four vials containing 2–3 million cells per CM formulation to be frozen. Viability and cell numbers were assessed by Trypan blue exclusion immediately after tumor dissociation and at both recovery time points (Fig. 5a). Decrease in cell viability upon cryopreservation was evident for both RCC and CRC samples (Fig. 5a). The commonly used cryopreservation medium, CM1, containing 90 FBS and 10% tissue grade neat DMSO, was the most effective in preserving cell viability for both tumor types, with approximately 80% of viable cells for both time points. In contrast, samples frozen with 90 FBS and 10% of glycerol (CM3) had viability below 60% at both time points for both tumor types. CM2 formulation contained conditioned media in which the tumors were stored following excision until processing, and the viability for both time points was above 70% at T1 and T2 for both tumor types. Under this condition, the viability measured for the RCC sample was 81.8 and 81.1%, while for the CRC samples the measured viability was 72.2 and 75.0% at T1 and T2 respectively. Because commercial AQIX media is optimized for preservation of cells and tissue biopsies [63], it is possible that the ability of this media to maintain pH levels at fluctuating temperatures [64], as well as the tissue specific growth factors secreted by cells while in transit, are responsible for preserving cellular viability. CM4, a serum-free, DMSO-free commercial media supplemented with a proprietary cryoprotectant was marked by a decrease in viability for the CRC sample measuring values at T1 of 76.7 and T2 of 66.7%. The viability of the RCC sample as compared to CRC was better with 74.3 viability at T1, and 88.0% at T2.

Although cell viability was best maintained with CM1 media, there still was a significant loss in total cell numbers as observed during both recovery time points. For the RCC sample, close to 90% of cells were lost from T1 to T2 post-freeze thaw time point as compared to other CM formulations. The CRC sample appeared to be more stable during cryopreservation, and cell recovery varied among the different media tested. The greatest decline in cell recovery from first to second thaw was observed with CM4, showing a 94% cell loss (Fig. 5a), while total cell loss measured for other cryomedia formulations was between 65 and 80%.

In addition to cell viability, we examined cryopreservation effects on the expression of common cell surface markers (Fig. 5b). For this analysis, raw median intensities for all surface markers were assessed in the CD45+ cell population. A representation of the different effects of cryopreservation on surface marker expression is depicted as histogram overlays (Fig. 5b). Our data indicates that 24 out of 39 analyzed markers show decreased median intensities upon cryopreservation. The majority of the markers affected by cryopreservation are those used to identify cells of myeloid lineage, such as CD11B, CD14, CD15, CD16, CD66B, CD80, CD86, and CD141. Additionally CD107a and CD25 expression levels are also decreased after cryopreservation. Both of these markers are associated with cellular activation, resulting in functional phenotypes implicated in anti-tumor response [65, 66]. Although most surface markers tested in this study are affected at varying levels, samples preserved in CM1 showed greater median intensities as compared to other cryomedia formulations (Fig. 5b). Expression patterns seen with CM3 had the least agreement with fresh samples. These findings are in alignment with the viability and cell number recovery measurements (Fig. 5a). Considering all aspects of cryopreservation, these data demonstrate that commonly used 90 FBS-10% DMSO formulation, although superior at preserving cellular viability as compared to other CM formulations, still has detrimental effects on expression levels of many surface markers.

To further determine if the observed expression level differences of several cell surface markers between frozen and fresh samples are associated with particular cell subpopulations, we applied a ViSNE analysis using the fresh RCC CM1 frozen sample (Fig. 5c). The resulting scatter plots show differential expression of multiple markers in fresh and frozen samples, thus altering spatial relationships of cells as determined by their phenotypes. While data indicates that Th-cells, Tc-cells and monocytes subpopulations are well identified in frozen cell preparations as compared to fresh samples, it also clearly highlights a specific loss of MDSCs as defined by co-expression of CD66b^+^ and CD15^+^, HLA-DR^dim^ and CD14^−^ phenotype. Similar results have been reported in whole blood sample analysis [62]. Because MDSCs are believed to be involved in regulation of tumor progression [67], preferential decrease in detection of this subpopulation might reduce the value of frozen tumor samples for biomarker research. In addition to common cell surface markers, we have evaluated expression of known immunoregulatory receptors on Th and Tc cell subsets (Additional file 3). Most noticeably, the median intensities of PD-1, its ligands PD-L1 and PD-L2, GITR and Lag-3 are significantly decreased in both T- cell subtypes upon cryopreservation. The observed decrease in median intensity ranged from 2- to 5-fold in all frozen samples, which is in agreement with previous publication using PBMCs [68]. Our results warrant further studies in identifying new cryopreservation media suitable for preserving multiple cellular phenotypes.

## Discussion

The ability to quantify effects of therapeutic intervention in heterogeneous cell populations and to correlate these with clinical outcome is of critical importance for the success of drug discovery and development. However, efficacy data from preclinical studies using animal models or immortalized cell lines does not always translate to clinical efficacy [69, 70], nor does it recapitulate the complex interactions responsible for cellular homeostasis. Simultaneous and quantitative measurement of multiple biomarkers that directly reflect cellular functional status in individual, primary patient cells, is, therefore, highly desired, particularly in immuno-oncology, where a deeper understanding of the complex responses of immune cells to tumors can facilitate discovery of new therapeutics and aid in patient stratification strategies. Mass cytometry, although relatively new to the field of single-cell analysis, has attracted significant interest for its ability to simultaneously profile up to 100 phenotypic and functional markers, enabling in-depth understanding of biomarker complexity [71, 72].

While several landmark studies have shown proof-of-concept [25, 43, 73, 74] of the MC analysis, comprehensive comparison to the gold standard of single cell analysis, flow cytometry, as applied to different samples, including tumors, is still limited. The initial analysis of frozen PBMC single donor sample over 9 independent runs enabled us to systematically evaluate precision and accuracy of analytical performance of the MC platform. The results of this study were in agreement with previously published data [73], and demonstrated that the major immune subsets constituting PBMCs are consistently detected using MC, with inter-run variability for all PBMC subpopulations below two-fold difference across nine runs.

Although we observed significant correlation between FC and MC using PBMC samples, the agreement between the two platforms using frozen tumor samples was variable. A number of factors can be considered to explain discrepancy for several tumor samples. Our data indicate that the samples exhibiting substantial discrepancy were marked by significant differences in acquired cell numbers and differences in viability determinations between FC and MC. Additionally the use of multiple panels required for FC, and overall lower sampling efficiencies reported for MC [73] played potential factors which contributed to variability in detected marker frequencies in both platforms. In contrast, tumor samples exhibiting significant correlation between FC and MC analyses had similar detected viability and acquired cell numbers on either platform. These data indicate that both FC and MC platforms are sensitive to quality and quantity of the analyzed samples, specifically evidenced with the commonly used frozen tumor samples.

To assess applicability of the MC platform for biomarker discovery in clinically relevant specimens, a comprehensive phenotyping analysis of fresh renal carcinoma using 39 simultaneous markers, validating detection of immune cell subtypes and various tumor cell subpopulations, was performed. The high multiplexing capability of CyTOF™ enabled identification of major leukocyte populations, as evaluated by the co-expression of various checkpoint regulators, as well as several tumor subtypes. For example, we were able to identify tumor subpopulations marked by presence of CD34^bright^ cells, potentially representing cancer stem cells (CSC), implicated in tumor initiation and progression [75]. However given significant heterogeneity reported in CSCs [76], it is impossible to definitively identify these cells as CSC without including additional markers known to be expressed on tumor initiating cells [77] or performing *in-vitro* experiments assessing functional pluripotent responses of these cells [78]. Regardless of the precise cellular identity of this population, the co-expression of CD107a (Lysosome Associated Membrane Protein-1, LAMP1) might suggest a connection to autocytolitic activity of NK cells [79]. We identified additional subpopulations marked by expression of CCR9 which potentially represent a subtype of tumor cells in the process of migrating to the small intestine where the CCR9 ligand, CCL25, is expressed [80, 81]. Furthermore, co-expression of inhibitory molecules CTLA-4, PD-L1 and PD-L2 on these tumor subtypes indicates the complex biology of tumor cells [82], suggesting that targeting multiple checkpoints expressed in particular tumors might have an additive therapeutic benefit. The phenotyping results of fresh clinical biospecimens confirm that these samples present a suitable model for understanding cancer pathophysiology.

As the end goal of this study to explore the use of MC analysis for clinical specimens, we further examined effect of cryopreservation on colon and renal cell carcinoma using four commonly used cryomedia formulations. Detrimental effects on both viability and cellular recovery were apparent using all media formulations, however the traditionally used freezing media of 90 FBS and 10% DMSO, was superior as compared to others, possibly due to DMSO’s ability to penetrate cells better than glycerol [83]. Extensive publications documenting detrimental effects of cryopreservation on cells and in particular embryonic stem cells [84, 85] could potentially explain the dramatic cell loss observed in this study. Because enzymatic digestion and mechanical dissociation have been implicated as the major contributing factors in inducing cellular apoptosis upon freezing [86, 87], similar effects, as a result of tissue processing and cryopreservation, may cause the observed decrease in DTC cell numbers. Further studies are required to determine if the observed differences in cryopreservation and recovery are organ and specimen specific, or are due to the sample processing methods.

In addition, cryopreservation affected the expression of many myeloid surface markers, possibly explaining the lack of detection of MDSC as previously described in PBMCs [62, 88]. Furthermore the decreased detection of CD107a and CD25 is particularly concerning as both markers are used to asses cellular activation states, as well as identification of CD25+ Treg cells [9], a subset critical for regulating anti-tumor immune response [89]. Our findings are also in agreement with previously published data documenting a damaging cryopreservation effects on PD-1 and PD-L1 detection in PBMCs [68], and further extend these results to tumor samples.

## Conclusion

In summary, our data suggests that results generated by MC are comparable to FC for both PBMC and tumor samples. However, MC analysis offers an improved ability for multiplexing of up to 39 markers. The obvious advantage of highly multiplexed MC capabilities is exemplified by the detection of tumor cells expressing markers potentially valuable for diagnosis, prognosis, assessment of treatment efficacy and patient stratification strategies. As the sensitivity and throughput are further improved of this still-developing platform, it is conceivable that MC could become the primary detection method for interrogation of complex interactions between tumor and immune subtypes at real time from clinical biopsies. This can be particularly critical in developing novel immunomodulatory therapies which are aimed at overcoming cancer resistance [90–92]. However, our data also cautions to the quality of frozen specimens used for biomarker discovery. The loss of specific subpopulations, particularly of those implicated in tumor-related biology, presents a challenge for using frozen clinical specimens for immunomodulatory biomarker studies. While additional studies extending to multiple tumors from various organ sources are needed to further corroborate these findings, the present study with renal and colon carcinomas supports further investment in developing more suitable clinical sample handling.

## Additional files

Additional file 1: Supplementary Table 1 provides a list of MC antibodies used for immunophenotyping of fresh and frozen RCC and CRC samples. (DOCX 75 kb)
Additional file 2: Supplemental data for Fig. 4 presents expression patterns of immunomodulatory and disease prognostic biomarkers monitored on immune and tumor cell subsets in fresh CRC as delineated by MC analysis. ViSNE analysis of fresh CRC performed using singlet live cells as top level population. A total of 10,902 events were sampled with cellular clustering performed using CD45, CD3, CD4, CD8a, CD20, CD56, CD11B, CD14, CD11C, and CD16 cell surface markers. Median expression levels of the markers listed in upper right corner of each plot is used for identification of cellular subtypes present in this sample. Expression of CD194 and CD183 on CD45^−^ cells are considered as potential clinical biomarkers, correlating with an advanced disease state and associating with significantly poorer prognosis and an increased metastatic potential of colorectal cancers. (DOCX 785 kb)
Additional file 3: Supplemental data for Fig. 5b provides additional information on cryopreservation effects on IMR expression in primary RCC samples. The data is depicted as histogram overlays of median intensities for selected markers as expressed in Th and Tc-cell subtypes. (DOCX 312 kb)

## Abbreviations

Ab: Antibody
ACK: Ammonium-chloride-potassium
AML: Acute myeloid leukemia
BSA: Bovine Serum Albumin
BTLA: B-and T-lymphocyte attenuator
CCL25: C-C motif chemokine ligand 25.
CCR9: C-C motif chemokine receptor 9
CM: Cryopreservation medium
CRC: Colorectal carcinoma
CSC: Cancer stem cells
CSM: Cell-staining-medium
CTLA-4: Cytotoxic T-lymphocyte antigen-4
CyTOF: Cytometry by time-of-flight
DC: Dendritic cells
DMSO: Dimethyl sulfoxide
DPBS: Dulbecco’s Phosphate-buffered Saline
DTC: Dissociated tumor cells
FBS: Fetal bovine serum
FC: Fluorescent cytometry (flow cytometry)
FMM: Flourescent minus multiple
GITR: Glucocorticoid-induced TNFR-related protein
HTS: High-throughput sampler
IMR: Immunoregulatory receptors
Lag-3: Lymphocyte-activation gene 3
LAMP-1: Lysosomal-associated membrane protein 1
MC: Mass cytometry
MDSC: Myeloid derived suppressor cells
MMM: Metal minus multiple
MS: Mass spectrometry
NAT: Normal adjacent tissue
NCAM: Neural cell adhesion molecule
NK-cells: Natural-killer cells
PBMC: Peripheral blood mononuclear cells
PD-1: Programmed cell death-1
PDL-1: Programmed cell death ligand-1
PDL-2: Programmed cell death ligand 2
PPMC: Pearson product moment correlation
RBC: Red blood cells
RCC: Renal cell carcinoma
RPMI medium: Roswell Park Memorial Institute medium
RT: Room temperature
Sp: Subpopulation
T1 and T2: Time point 1 and time point 2
Tc: T-cytotoxic cells
Th: T-helper cells
TIL: Tumor infiltrating lymphocyte
TIL: Tumor infiltrating lymphocyte
Treg: T-regulatory cells
