# Supplementary figures and images for: Effect of cryopreservation on delineation of immune cell subpopulations in tumor specimens as determinated by multiparametric single cell mass cytometry analysis
Source: BMC Immunol. 2017 Feb 2;18:6. doi: 10.1186/s12865-017-0192-1 (PMC5288879; doi:10.1186/s12865-017-0192-1)

**Additional File 1.**

**
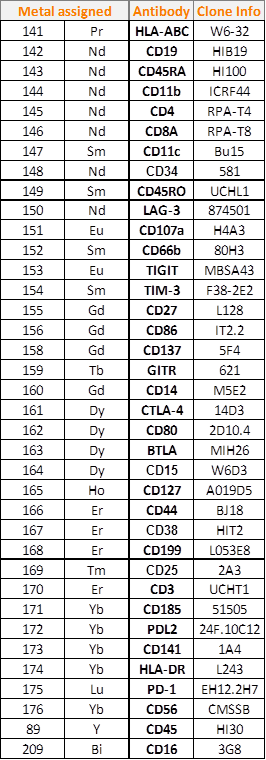
**

Supplement: Additional file 1: — Supplementary Table 1 provides a list of MC antibodies used for immunophenotyping of fresh and frozen RCC and CRC samples. (DOCX 75 kb) [file 12865_2017_192_MOESM1_ESM.docx]

**Additional File 2.**

**
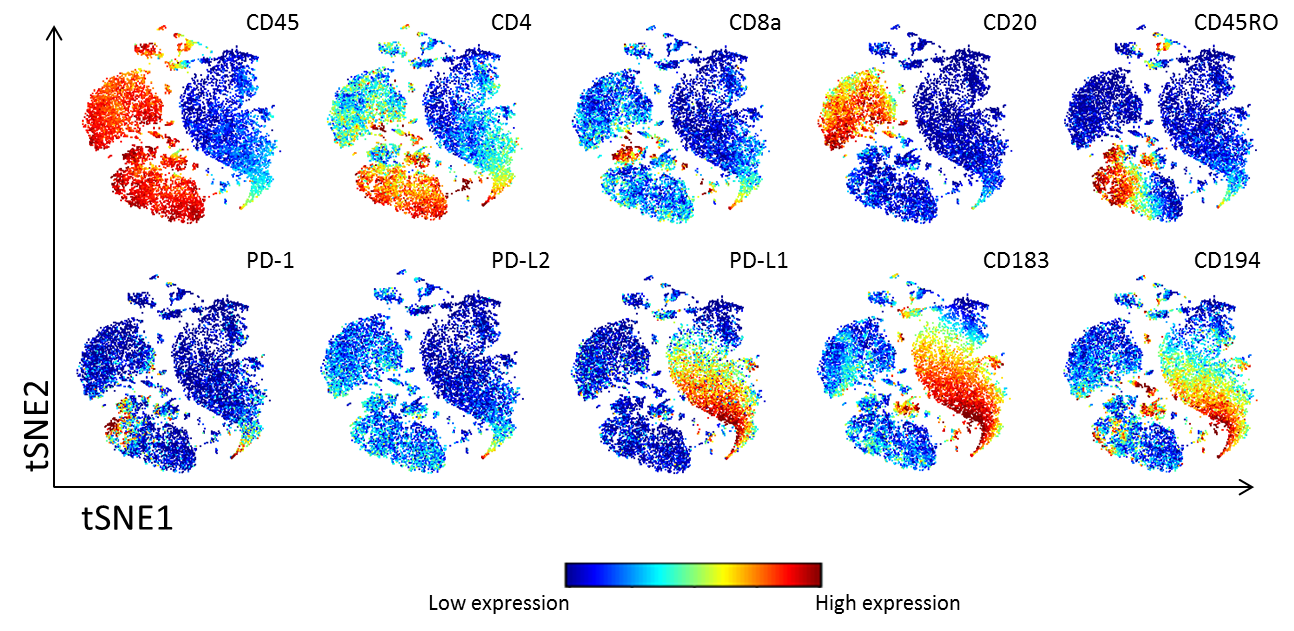
**

Supplement: Additional file 2: — Supplemental data for Fig. 4 presents expression patterns of immunomodulatory and disease prognostic biomarkers monitored on immune and tumor cell subsets in fresh CRC as delineated by MC analysis. ViSNE analysis of fresh CRC performed using singlet live cells as top level population. A total of 10,902 events were sampled with cellular clustering performed using CD45, CD3, CD4, CD8a, CD20, CD56, CD11B, CD14, CD11C, and CD16 cell surface markers. Median expression levels of the markers listed in upper right corner of each plot is used for identification of cellular subtypes present in this sample. Expression of CD194 and CD183 on CD45− cells are considered as potential clinical biomarkers, correlating with an advanced disease state and associating with significantly poorer prognosis and an increased metastatic potential of colorectal cancers. (DOCX 785 kb) [file 12865_2017_192_MOESM2_ESM.docx]

**Additional File 3.**

**
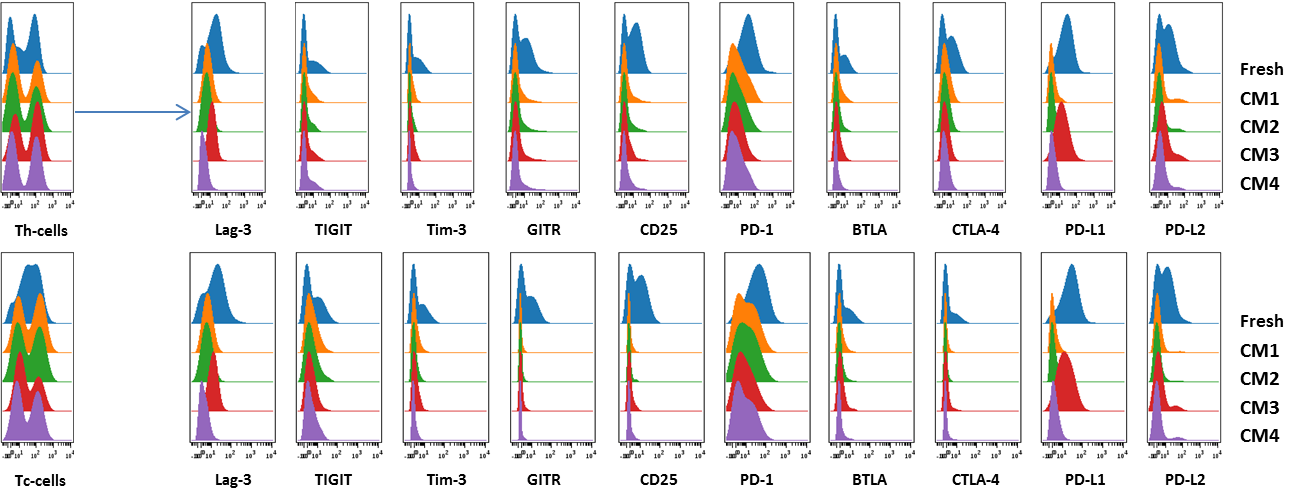
**

Supplement: Additional file 3: — Supplemental data for Fig. 5b provides additional information on cryopreservation effects on IMR expression in primary RCC samples. The data is depicted as histogram overlays of median intensities for selected markers as expressed in Th and Tc-cell subtypes. (DOCX 312 kb) [file 12865_2017_192_MOESM3_ESM.docx]
